# Clinical biomarker‐based biological aging and risk of benign prostatic hyperplasia: A large prospective cohort study

**DOI:** 10.1002/agm2.12331

**Published:** 2024-06-14

**Authors:** Qiao Huang, Bing‐Hui Li, Yong‐Bo Wang, Hao Zi, Yuan‐Yuan Zhang, Fei Li, Cheng Fang, Shi‐Di Tang, Ying‐Hui Jin, Jiao Huang, Xian‐Tao Zeng

**Affiliations:** ^1^ Center for Evidence‐Based and Translational Medicine Zhongnan Hospital of Wuhan University Wuhan China; ^2^ Department of Evidence‐Based Medicine and Clinical Epidemiology, Second School of Clinical Medicine Wuhan University Wuhan China; ^3^ Department of Urology Zhongnan Hospital of Wuhan University Wuhan China; ^4^ Department of Geriatrics Zhongnan Hospital of Wuhan University Wuhan China; ^5^ Department of Epidemiology and Biostatistics, School of Health Sciences Wuhan University Wuhan China

**Keywords:** accelerated age, aging, benign prostatic hyperplasia, biological age, chronological age

## Abstract

**Objective:**

Chronological age (CAge), biological age (BAge), and accelerated age (AAge) are all important for aging‐related diseases. CAge is a known risk factor for benign prostatic hyperplasia (BPH); However, the evidence of association of BAge and AAge with BPH is limited. This study aimed to evaluate the association of CAge, Bage, and AAge with BPH in a large prospective cohort.

**Method:**

A total of 135,933 males without BPH at enrolment were extracted from the UK biobank. We calculated three BAge measures (Klemera–Doubal method, KDM; PhenoAge; homeostatic dysregulation, HD) based on 16 biomarkers. Additionally, we calculated KDM‐BAge and PhenoAge‐BAge measures based on the Levine method. The KDM‐AAge and PhenoAge‐AAge were assessed by the difference between CAge and BAge and were standardized (mean = 0 and standard deviation [SD] = 1). Cox proportional hazard models were applied to assess the associations of CAge, Bage, and AAge with incident BPH risk.

**Results:**

During a median follow‐up of 13.150 years, 11,811 (8.690%) incident BPH were identified. Advanced CAge and BAge measures were associated with an increased risk of BPH, showing threshold effects at a later age (all *P* for nonlinearity <0.001). Nonlinear relationships between AAge measures and risk of BPH were also found for KDM‐AAge (*P* = 0.041) and PhenoAge‐AAge (*P* = 0.020). Compared to the balance comparison group (−1 SD < AAge < 1 SD), the accelerated aging group (AAge > 2 SD) had a significantly elevated BPH risk with hazard ratio (HR) of 1.115 (95% CI, 1.000–1.223) for KDM‐AAge and 1.180 (95% CI, 1.068–1.303) for PhenoAge‐AAge, respectively. For PhenoAge‐AAge, subgroup analysis of the accelerated aging group showed an increased HR of 1.904 (95% CI, 1.374–2.639) in males with CAge <50 years and 1.233 (95% CI, 1.088–1.397) in those having testosterone levels <12 nmol/L. Moreover, AAge‐associated risk of BPH was independent of and additive to genetic risk.

**Conclusions:**

Biological aging is an independent and modifiable risk factor for BPH. We suggest performing active health interventions to slow biological aging, which will help mitigate the progression of prostate aging and further reduce the burden of BPH.

## Graphical abstract

Biological aging is an independent and modifiable risk factor for BPH. Performing active health interventions to slow biological aging, which will help mitigate the progression of the prostate aging.

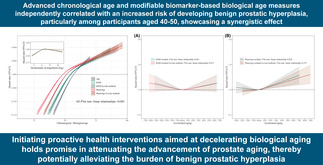

## 1 INTRODUCTION

Chronological age (CAge), calculated as the duration since birth, provides a convenient way to quantify individuals' aging state. Aging is a heterogeneous process, and individuals of the same CAge can exhibit great variation in health outcomes.^1^, ^2^ Hence, biological age (BAge) and accelerated age (AAge) were introduced. BAge, a comprehensive measure assessing an individual's physiological state, has been proposed as a potentially more accurate indicator for evaluating the age‐related risk of adverse outcomes.^3^ AAge is defined as the disparity between biological and chronological age. In recent years, various algorithms have been utilized to measure BAge and AAge, using a range of indicators, including phenotypic indicators, omics‐based indicators, and clinical indicators.^4^ Among them, clinical indicators have the advantage of detecting physiological changes at an earlier stage compared to specific phenotypes. Increasing evidence supports associations between BAge and aging‐related diseases, including neurodegenerative diseases, cardiovascular diseases, cancers, and musculoskeletal disorders^5^, ^6^, ^7^, ^8^; for instance, BAge was found to be a better predictor of 3‐month outcomes in ischemic stroke compared to CAge.^9^

Aging is a major risk factor for prostate aging and benign prostatic hyperplasia (BPH) development and can be characterized in chronological or biological dimensions.^10^ Prostate aging and its development to BPH commonly occurs from the age of 40 years.^11^ BPH is a global challenge for aging males.^12^ The prevalence of BPH is as high as 50% for males whos CAge in their 50s, increasing to 80% for those aged 80 years or older.^10^ However, the impact of biological aging on the development of BPH has not yet been established. Therefore, we assessed the relationships between different BAge measures and the risk of BPH using the UK Biobank.

Since different types of biological age measures capture distinct aspects of the aging process, we adopted three validated algorithms (Klemera–Doubal method [KDM], PhenoAge, and homeostatic dysregulation [HD]).^13^ We explored the effect of AAge on the risk of incident BPH. Furthermore, we performed secondary analyses to examine the potential interactions between AAge and basic characteristics, including CAge, testosterone levels, and genetic risk for BPH. Validating the association between CAge, BAge, Aage, and BPH could provide potential opportunities for targeting biological aging pathways. In turn, this may facilitate the development of effective interventions to mitigate the occurrence and progression of BPH and global aging.

## 2 METHODS

### 2.1 Study design and population

The UK Biobank is a large‐scale and continuously evolving longitudinal cohort study approved by the UK North West Multicenter Research Ethical Committee.^14^ It has recruited 229,071 males aged between 38 and 73 years at baseline during the period from 2006 to 2010. Prior to participation, all individuals provided written informed consent. Baseline assessments, physical examination, and collection of biological samples were conducted in 22 assessment centers throughout England, Wales, and Scotland. Health‐related outcomes have been acquired by conducting periodic linkages to various national databases. In this study, two individuals withdrew consent and one individual reported an invalid date on BPH. Additionally, 14,584 participants were confirmed as having BPH at baseline or during the 6‐month follow‐up period; Furthermore, 3245 participants were diagnosed with prostate cancer at baseline, while 823 participants had been diagnosed with prostate cancer before being diagnosed with BPH during the follow‐up period. Ultimately, a total of 210,416 participants were included for next analysis (Figure S1).

### 2.2 Assessment of biological and accelerated ages

In this study, we employed widely recognized and validated algorithms to assess BAge, namely the KDM,^15^ PhenoAge,^16^ and HD,^17^ which have been extensively summarized and validated.^13^, ^18^ First, data from the US National Health and Nutrition Examination Surveys (NHANES) III without missing data in required biomarkers were used as a reference for the estimation of algorithm parameters. Notably, the algorithm parameters are estimated separately for males and females. Second, the BAge algorithms were validated using the NHANES IV database. Finally, the UK Biobank data were utilized to project BAge, facilitating further analysis.

In KDM‐BAge estimation, participants in NHANES III aged 30–75 years were used as the reference. The KDM‐BAge was determined by separate regressions of CAge against a set of *n* biomarkers in the reference group. The estimated BAge of UK Biobank data was described as the age at which an individual's physiology aligns with the average physiology observed in the reference. The reference in PhenoAge included participants aged 20–84 years. The PhenoAge algorithm uses the elastic‐net Gompertza regression of mortality to project mortality prediction scores based on biomarkers and CAge. The PhenoAge is described as the age at which the predicted mortality risk in the UK Biobank dataset corresponds to the average mortality risk observed in the NHANES III dataset. The HD algorithm estimated the Mahalanobis distance between the reference and the projection population instead of age. The reference group for HD included participants aged 20–30 years, who met the criteria of not being obese and having biomarker values within age‐ and sex‐specific normal ranges. The HD algorithm provides an estimation of how an individual's physiology deviates from that of a healthy sample observed in the NHANES III dataset.

In the three algorithms, any age‐related biomarkers can be employed in the construction of BAge. In accordance with recent publications, we selected 19 potential biomarkers covering a range of the human organ systems, which were available in both NHANES and UK Biobank databases.^6^, ^19^ Furthermore, the Levine method adopted some of the 19 biomarkers for quantifying biological aging, KDM‐BAge (Levine method) and PhenoAge‐BAge (Levine method) were calculated for comparison.^16^ Based on previous studies, we excluded diastolic blood pressure (DBP) and uric acid from the projection of BAge among our male participants due to their weak correlation with CAge (|*r*| < 0.1).^6^, ^20^ Detailed information on the biomarkers used for BAge calculation and their measurement methods are provided in Tables S1 and S2.

The BAges were calculated using the R package “BioAge.”^13^ Participants with missing data in the CAge (*n* = 2), 19 biomarkers (*n* = 69,899), and outlier BAge values (defined as values less than −5 or more than +5 standard deviation [SD] from the mean) (*n* = 77) were excluded (Figure S1). Regressions of the BAge measures (KDM and PhenoAge) on CAge using 3 degrees‐of‐freedom natural spline were conducted, the AAge was quantified by calculating the regression residuals, which gave KDM‐AAge, KDM‐AAge (Levine method), PhenoAge‐AAge and PhenoAge‐AAge (Levine method).^21^ To enable comparison of effect sizes in different algorithms, the residuals were transformed into *Z*‐scores (mean = 0 and SD = 1). Higher *Z*‐score values indicated a more advanced acceleration. The *Z*‐scores were further classified into five levels: [min, −2 SD), [−2 SD, −1 SD), [−1 SD, 1 SD], (1 SD, 2 SD], (2 SD, max].

### 2.3 BPH ascertainment and its polygenic risk scores

The UK biobank reported the diagnoses of BPH (Data‐Field 132073) and corresponding time (Data‐Field 132072). For participants free of BPH at baseline or during the 6‐month follow‐up period, their follow‐up time ended on the date of incident BPH (at least 6‐month follow‐up), loss to follow‐up (Data‐Field 191), death (Data‐Field 40000), or end of study (July 1, 2022), whichever occurred first.

In this study, we identified 19 independent single nucleotide polymorphisms (SNPs) that showed significant genome‐wide association (*p* < 5e^−8^) with BPH in published genome‐wide association studies (GWASs).^22^, ^23^ Details regarding the selected SNPs are provided in Table S3. Individual SNP is recoded as 0, 1, and 2 according to the number of risk alleles. The polygenic risk score (PRS) for BPH was calculated based on the published method, where weighted PRS = (*β*_1_ × SNP_1_ + *β*_2_ × SNP_2_ + …… + *β*_*n*_ × SNP_*n*_)/(2 × *n*).^24^ We determined whether participants were at high (> median of PRS) or low (≤ median of PRS) genetic risk based on their genetic profile.

### 2.4 Measurements of covariates

We included age, assessment centers (Scotland/Wales/England), the Townsend deprivation index (TDI, continuous), college/university degree (Yes/No), ethnicity (White/Asian/Black/Others), body mass index (BMI), testosterone, smoking status (Never/Previous/Current), alcohol status (Never/Previous/Current), regular physical activity status (Ideal/Intermediate or poor/unknown), sedentary status (Ideal/Intermediate or poor/unknown), sleep status (Ideal/Intermediate or poor/unknown) and diet status (Ideal/Intermediate or poor/unknown) as potential covariates. We divided the age into three groups: <50, 50–60, and >60 years. TDI was divided into four classes based on quantiles <−3.63, −3.63 to ≤−2.09, −2.09 to 0.68, and >0.68. BMI was classified into: underweight (<18.5 kg/m^2^), normal weight (18.5 to <25), overweight (25 to <30), and obese (≥30). Regular physical activity was assessed based on the weekly duration of moderate and vigorous activities. The sleep index was determined by sleep duration, morning routine, chronotype, sleeplessness/insomnia, snoring, and daytime dozing or sleeping. Sedentary status was calculated using the time spent on computer use and watching television. The diet pattern was evaluated by the intake of fruits, vegetables, fish, and processed and red meat. Details of defining the types (poor/intermediate/ideal/unknown) of status of physical activity, sedentary, sleep index, and diet pattern are documented in Table S4. Observations with any missing value of the covariates were excluded (*n* = 4505).

### 2.5 Statistical analyses

Descriptive statistics, including mean with SD for continuous variables and count with percentages for categorical variables, were used to summarize the characteristics of the participants. Student's *t*‐test was employed to assess differences between groups in continuous variables. The Chi‐square test was used to examine differences between groups in categorical variables.

We estimated and visualized incidence of BPH in different combinations of BAge and AAge groups with sample size. A series of Cox proportional‐hazards models were used to explore the relationship between CAge, Bage, and AAge and incidence of BPH. The selection of semi‐parametric Cox model was informed by several considerations: (1) the Cox model is well‐suited for time‐to‐event outcome, which is the nature of the BPH incidence data; (2) it allows for the inclusion of multiple covariates (both quantitative and qualitative factors) enabling us to simultaneously control for potential confounding factors; (3) and it provides hazard ratio (HR) as effect measure to assess the magnitude and directions of association over the entire length of follow‐up instead of single one‐time point. The proportional hazard assumption was formally tested for exposure of interest and no evidence of violation was found. This indicated that the results of the Cox PH model were robust. First, to flexibly model and visualize the relationship between CAge, Bage, and risk of incident BPH, we used multivariable Cox proportional‐hazards models with adjustment of covariates and restricted cubic spline with knots at the 10th, 50th, and 90th percentiles. Likelihood ratio tests (LRT) were used to test for potential nonlinearity and interaction effects. Second, for exploring the effect of AAge on risk of incident BPH, Cox proportional‐hazards models with attained age as the underlying timescale were adopted. HR and corresponding 95% confidence interval (CI) were reported. A simple age‐adjusted model was reported for continuous AAge. In multivariable model, continuous AAge (HRs per 1‐SD increase) and categorized AAge (−1 SD to 1 SD as reference) were analyzed separately with adjustment of other covariates. Covariates used in each model were listed in the footnote of corresponding tables. To examine potential nonlinear relationships, restricted cubic spline of AAge with knots at the 10th, 50th, and 90th percentiles was also applied with LRT. Lastly, sensitivity analyses were conducted. We performed subgroup analyses to test whether the associations between AAge and risk of BPH may differ by CAge at baseline (<50, 50–60, >60 years) and testosterone (<12 nmol/L, ≥12 nmol/L). To further explore the potential modification effect of AAge on CAge, we estimated the incidence of BPH in each combination of categorical CAge and categorical and AAge. Subsequently, we fitted a Cox model with restricted cubic spline for continuous CAge and its interaction with a three‐level AAge (<−2 SD, −2 SD to 2 SD, >2 SD) for simplification. Interaction effects were tested using LRT.

The relationship between PRS and Incident BPH was validated using the Cox model with full adjustment. To evaluate whether genetic predisposition to BPH may modify the association between AAge and risk of incident BPH, we fitted a model to test the interaction term between 5‐level AAge and dichotomous PRS using LRT. Meanwhile, a categorical variable combining the two categorical variables was created, and the joint effects were estimated and visualized using the combination of AAge from −1 SD to 1 SD and low gene risk group as reference.

All statistical analyses and data visualization were performed using the R program (version 4.0.3; R Core Team, Vienna, Austria). All tests were two‐tailed, with significance defined as *p* < 0.05.

## 3 RESULTS

### 3.1 Sample characteristics

A total of 135,933 participants were included for final analysis, the mean age at baseline was 56 years (ranging from 38 to 73 years); Of them, 91.873% were from England, 94.939% were white, and 94.176% were currently drinking alcohol (Table 1). During a median follow‐up of 13.150 years (interquartile range 12.293–13.873), a total of 11,811 incident BPH (8.689%) were found (Table 2). The incidences were 2.355% in <50 years' group, 7.780% in 50–60 years' group, and 14.159% in >60 years' group, respectively.

**TABLE 1:** Characteristics of study participants in UK Biobank.

Biomarkers	Overall (*n* = 135,933)	<50 years^a^ (*n* = 34,954)	50–60 years (*n* = 51,887)	>60 years^b^ (*n* = 49,092)	*P*
Assessment center, *n* (%)					
Scotland	5054 (3.718)	1359 (3.888)	2043 (3.947)	1652 (3.365)	<0.001
Wales	5993 (4.409)	1601 (4.580)	2459 (4.739)	1933 (3.938)	
England	125,000 (91.873)	31,994 (91.532)	47,385 (91.323)	45,507 (92.697)	
Townsend deprivation index, *n* (%)					
[−6.26, −3.63]	34,213 (25.169)	9106 (26.051)	13,150 (25.344)	11,957 (24.356)	<0.001
(−3.63, −2.09]	34,668 (25.504)	7928 (22.681)	13,277 (25.588)	13,463 (27.424)	
(−2.09, 0.68]	35,083 (25.809)	7725 (22.100)	13,472 (25.964)	13,886 (28.286)	
(0.68, 10.9]	31,969 (23.518)	10,195 (29.167)	11,988 (23.104)	9786 (19.934)	
College/university degree, *n* (%)					
No	88,808 (65.332)	21,176 (60.582)	32,572 (62.775)	35,060 (71.417)	<0.001
Yes	47,125 (34.668)	13,778 (39.418)	19,315 (37.225)	14,032 (28.583)	
Ethnicity, *n* (%)					
White	129,000 (94.939)	31,818 (91.028)	49,453 (95.309)	47,782 (97.332)	<0.001
Asian	3352 (2.466)	1376 (3.937)	1238 (2.386)	738 (1.503)	
Black	1718 (1.264)	886 (2.535)	595 (1.147)	237 (0.483)	
Others	1810 (1.332)	874 (2.500)	601 (1.158)	335 (0.682)	
Body mass index, kg/m^2^, *n* (%)					
<18.5	301 (0.221)	93 (0.266)	121 (0.233)	87 (0.177)	<0.001
18.5–24.9	32,970 (24.255)	9438 (27.001)	12,365 (23.831)	11,167 (22.747)	
25–29.9	67,996 (50.022)	17,159 (49.090)	25,582 (49.303)	25,255 (51.444)	
>29.9	34,666 (25.502)	8264 (23.643)	13,819 (26.633)	12,583 (25.631)	
Testosterone, nmol/L, mean ± SD	12.022 ± 3.677	12.410 ± 3.756	12.003 ± 3.648	11.765 ± 3.627	<0.001
Smoking status, *n* (%)					
Never	67,866 (49.926)	20,635 (59.03)	26,856 (51.76)	20,375 (41.50)	<0.001
Previous	51,220 (37.680)	8717 (24.94)	18,562 (35.77)	23,941 (48.77)	
Current	16,847 (12.394)	5602 (16.03)	6469 (12.47)	4776 (9.73)	
Alcohol status, *n* (%)					
Never	3488 (2.566)	1236 (3.536)	1078 (2.078)	1174 (2.391)	<0.001
Previous	4429 (3.258)	1125 (3.219)	1742 (3.357)	1562 (3.182)	
Current	128,000 (94.176)	32,593 (93.245)	49,067 (94.565)	46,356 (94.427)	
Physical activity, *n* (%)					
Ideal	58,545 (43.069)	16,723 (47.843)	21,639 (41.704)	20,183 (41.113)	<0.001
Intermediate/poor	17,017 (12.519)	5031 (14.393)	6863 (13.227)	5123 (10.436)	
Unknown	60,371 (44.412)	13,200 (37.764)	23,385 (45.069)	23,786 (48.452)	
Sedentary status, *n* (%)					
Ideal	4891 (3.598)	1287 (3.682)	2034 (3.920)	1570 (3.198)	<0.001
Intermediate/poor	102,000 (75.190)	24,861 (71.125)	38,199 (73.620)	39,148 (79.744)	
Unknown	28,834 (21.212)	8806 (25.193)	11,654 (22.460)	8374 (17.058)	
Sleep status, *n* (%)					
Ideal	37,739 (27.763)	9363 (26.787)	14,073 (27.120)	14,303 (29.135)	<0.001
Intermediate/poor	73,726 (54.237)	19,403 (55.510)	28,369 (54.675)	25,954 (52.868)	
Unknown	24,468 (18.000)	6188 (17.703)	9445 (18.203)	8835 (17.997)	
Diet pattern					
Ideal	51,358 (37.782)	11,331 (32.417)	19,063 (36.739)	20,964 (42.703)	<0.001
Intermediate/poor	59,389 (43.690)	16,507 (47.225)	23,034 (44.393)	19,848 (40.430)	
Unknown	25,186 (18.528)	7116 (20.358)	9790 (18.868)	8280 (16.866)	

Abbreviations: BPH, benign prostatic hyperplasia; DBP, diastolic blood pressure; FEV1, forced expiratory volume in 1 s; HbA1c, glycated hemoglobin; HD, homeostatic dysregulation; KDM, Klemera–Doubal method; RBC, red blood cell; SBP, systolic blood pressure; WBC, white blood cell.

^a^ Four subjects under the age of 40, the minimal age is 38.

^b^ Four subjects over the age of 70, the maximal age is 73.

**TABLE 2:** Biological age, accelerated aging of study participants in UK Biobank.

Biomarkers	Overall (*n* = 135,933)	<50 years^a^ (*n* = 34,954)	50–60 years (*n* = 51,887)	>60 years^b^ (*n* = 49,092)	*P*
Incident BPH, *n* (%)	11,811 (8.689)	823 (2.355)	4037 (7.780)	6951 (14.159)	<0.001
Chronological age at baseline (year)	56.002 ± 8.188	44.87 ± 2.76	55.364 ± 3.206	64.604 ± 2.583	<0.001
Biological age at baseline (year)					
KDM‐BAge (new method)	53.862 ± 9.209	43.231 ± 5.238	53.105 ± 5.647	62.232 ± 5.516	<0.001
KDM‐BAge (Levine method)	54.235 ± 8.955	43.526 ± 4.774	53.456 ± 5.132	62.683 ± 4.931	<0.001
PhenoAge‐BAge (new method)	47.965 ± 10.483	36.000 ± 5.941	47.010 ± 6.454	57.494 ± 6.439	<0.001
PhenoAge‐BAge (Levine method)	50.720 ± 9.708	39.003 ± 4.926	49.935 ± 5.460	59.891 ± 5.448	<0.001
HD (log units)	6.342 ± 0.997	6.153 ± 1.056	6.336 ± 0.997	6.481 ± 0.928	<0.001
Accelerated age					
KDM‐AAge (new method)	−0.039 ± 4.752	−0.066 ± 4.586	−0.027 ± 4.774	−0.032 ± 4.844	0.453
KDM‐AAge (Levine method)	−0.033 ± 4.113	−0.061 ± 4.047	−0.021 ± 4.120	−0.026 ± 4.152	0.330
PhenoAge‐AAge (new method)	−0.043 ± 5.487	−0.083 ± 5.272	−0.029 ± 5.470	−0.030 ± 5.652	0.300
PhenoAge‐AAge (Levine method)	−0.019 ± 4.359	−0.028 ± 4.028	−0.018 ± 4.309	−0.015 ± 4.629	0.897
Biomarkers					
FEV_1_ (L)	3.340 ± 0.764	3.741 ± 0.726	3.391 ± 0.711	3.000 ± 0.682	<0.001
Waist circumference (cm)	96.690 ± 11.189	94.675 ± 11.064	96.980 ± 11.360	97.818 ± 10.900	<0.001
Total cholesterol (mg/dL)	213.597 ± 43.350	217.999 ± 41.088	217.263 ± 42.864	206.587 ± 44.525	<0.001
Triglyceride (mg/dL)	175.125 ± 101.497	178.790 ± 110.921	177.696 ± 102.645	169.798 ± 92.651	<0.001
SBP (mm Hg)	142.477 ± 18.324	135.883 ± 15.410	141.749 ± 17.659	147.943 ± 19.227	<0.001
HbA1c (%)	5.467 ± 0.660	5.321 ± 0.599	5.466 ± 0.669	5.573 ± 0.670	<0.001
Serum glucose (mmol/L)	5.164 ± 1.347	4.992 ± 1.215	5.160 ± 1.384	5.290 ± 1.382	<0.001
Blood urea nitrogen (mg/dL)	15.611 ± 3.850	14.771 ± 3.452	15.407 ± 3.707	16.425 ± 4.101	<0.001
C‐reactive protein (mg/dL)	0.232 ± 0.398	0.220 ± 0.353	0.225 ± 0.383	0.260 ± 0.439	<0.001
Creatinine (μmol/L)	81.443 ± 14.240	80.282 ± 12.726	80.828 ± 13.711	82.920 ± 15.620	<0.001
Albumin (g/dL)	45.652 ± 2.577	46.517 ± 2.504	45.669 ± 2.494	45.018 ± 2.533	<0.001
Alkaline phosphatase (U/L)	81.549 ± 23.680	80.372 ± 21.741	81.366 ± 23.764	82.580 ± 24.843	<0.001
Red cell distribution width (%)	13.415 ± 0.837	13.261 ± 0.757	13.397 ± 0.814	13.542 ± 0.895	<0.001
Mean cell volume (fL)	82.653 ± 5.287	81.740 ± 5.166	82.575 ± 5.200	83.387 ± 5.356	<0.001
Lymphocyte (%)	28.092 ± 7.381	29.205 ± 7.284	28.296 ± 7.245	27.088 ± 7.461	<0.001
RBC count (million cells/μL)	4.757 ± 0.374	4.829 ± 0.357	4.759 ± 0.365	4.702 ± 0.385	<0.001
WBC count (1000 cells/μL)	6.874 ± 1.885	6.675 ± 1.826	6.845 ± 1.862	7.046 ± 1.934	<0.001

Abbreviations: AAge, accelerated age; BAge, biological age; BPH, benign prostatic hyperplasia; DBP, diastolic blood pressure; FEV1, forced expiratory volume in 1 second; HbA1c, glycated hemoglobin; HD, homeostatic dysregulation; KDM, Klemera–Doubal method; RBC, red blood cell; SBP, systolic blood pressure; WBC, white blood cell.

^a^ Four subjects under the age of 40, the minimal age is 38.

^b^ Four subjects over the age of 70, the maximal age is 73.

**FIGURE 1:**
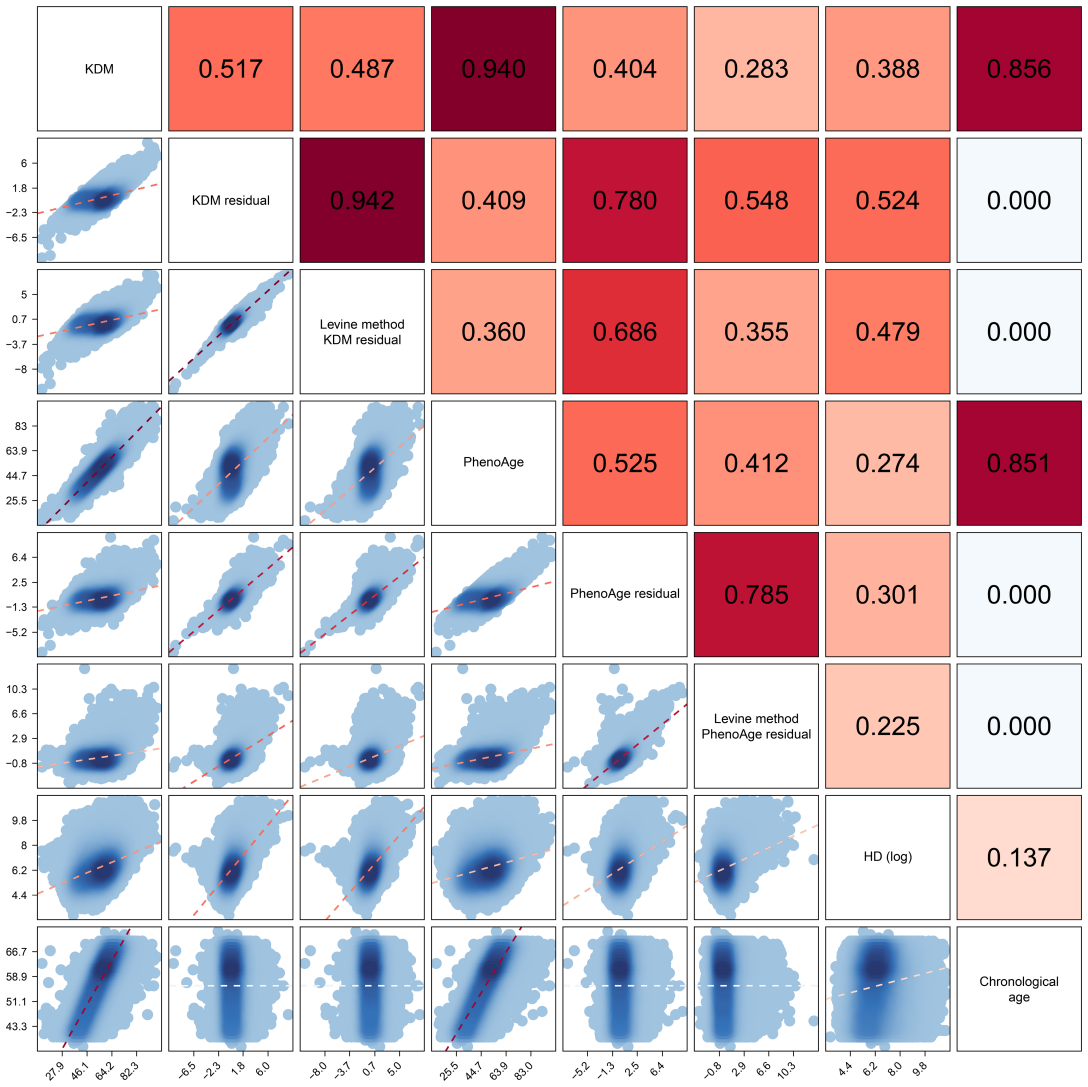
Correlations among chronological age, biological age, and accelerating age.

Description of CAge, BAge, Aage, and corresponding biomarkers are summarized in Table 2. The five BAge measures and all included biomarkers showed significant differences between the three CAge groups, however, no significant differences were observed in the standardized AAges. Correlations among CAge, BAge, and AAge are shown in Figure 1 and Figure S2. As expected, strong correlations were identified between CAge and KDM‐BAge (*r* = 0.856), as well as between CAge and PhenoAge (*r* = 0.851). However, the correlation between CAge and HD was weak (*r* = 0.137). After removing the CAge effect, four AAges (KDM‐AAge [new and Levine], PhenoAge‐AAge [new and Levine]) were not correlated with CAge (all *r* < 0.001). The KDM‐AAge (residual) was strongly correlated with PhenoAge‐AAge (residual) with a *r* = 0.780, while the *r* changed to 0.355 using the Levine method. The correlations between HD and four AAges ranged from 0.225 to 0.524.

### 3.2 Chorological and biological age, accelerated age, and risk of incident BPH

**FIGURE 2:**
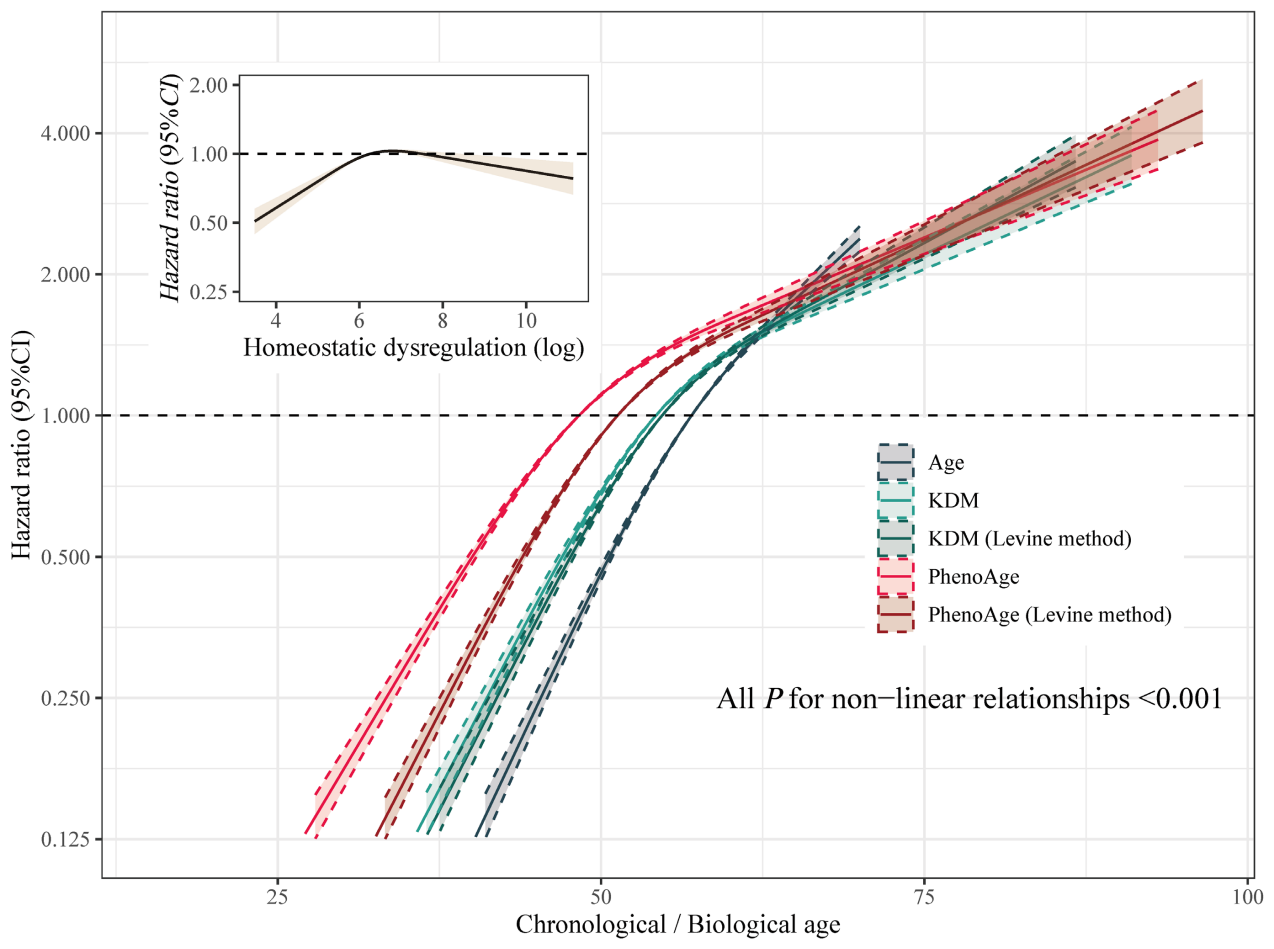
Chronological and biological age and risk of incident BPH at follow‐up.

Significant nonlinear relationships between CAge and 5 BAges and risk of incident BPH were found, with all *p* values for nonlinearity <0.001 (Figure 2). As the measures increased from low value, the risk of BPH showed a noticeable rise, but in later stages, the rate of risk increment slowed down. Among them, the threshold effect is evident in HD. Meanwhile, many of the 16 clinical biomarkers were also associated with BPH risk (Table S5). For instance, higher FEV1 was associated with decreased risk but higher HbA1c increased BPH risk.

**FIGURE 3:**
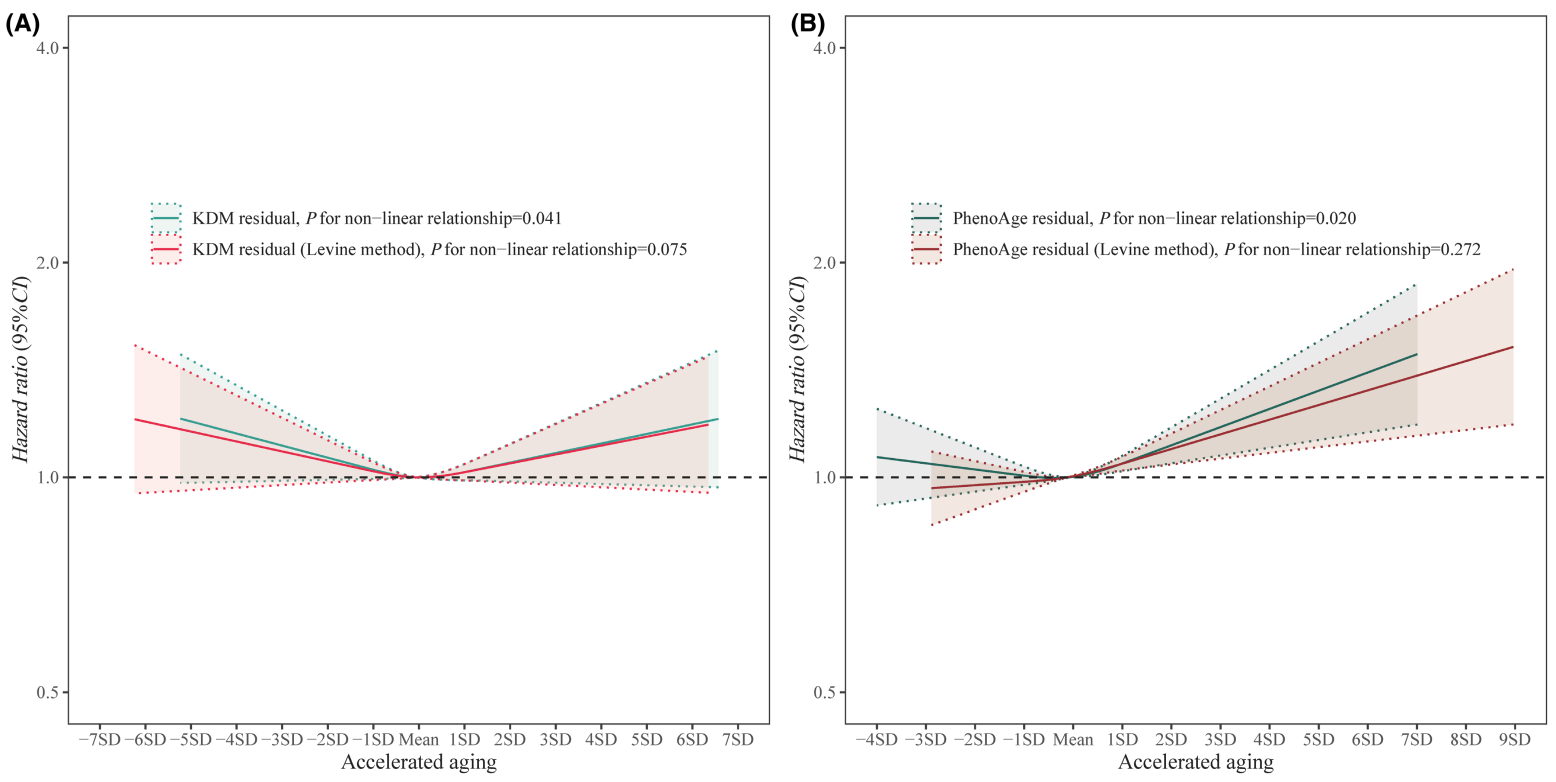
Accelerating age and risk of incident BPH at follow‐up.

In the age‐adjusted model, both KDM‐AAge (new and Levine) and PhenoAge‐AAge (new and Levine) were significantly associated with an elevated risk of BPH (Table 3). After full adjustment of covariates, only PhenoAge‐AAge remained the significant associations, HR per 1 − SD increase was 1.035 (95% *W*, 1.016–1.054) for the Levine method and 1.025 (95% CI, 1.006–1.045) for the new method, respectively. Nonlinear analysis and visualization supported statistically nonlinear relationships between AAges (new methods) and risk of BPH, *p* = 0.041 for KDM‐AAge and 0.020 for PhenoAge‐AAge (Figure 3). Compared with no accelerated age (AAge = 0), the risk of BPH increased significantly with advanced AAge (AAge > 0). Even though not statistically significant in the Levine methods, they showed similar trends to the new methods. It was validated in the multivariable model using categorical AAge (Table 3). When comparing the AAge group (>2 SD) with the balance aging group (−1 SD to 1 SD), we observed a significant increase in the risk of BPH, with a HR of 1.115 (95% CI, 1.000–1.223) for KDM‐AAge (new method), 1.119 (1.006–1.244) for KDM‐AAge (Levine method), 1.180 (1.068–1.303) for PhenoAge‐AAge (new method), and 1.155 (1.051–1.270) for PhenoAge‐AAge (Levine method).

**TABLE 3:** Association between accelerated aging and risk of incident BPH at follow‐up in UK Biobank.

Model	KDM–AAge			PhenoAge‐AAge		
	*n*	Hazard ratio (95% CI)	*P*	*n*	Hazard ratio (95% CI)	*P*
New method (16 biomarkers)						
Age‐adjusted model (continuous)^a^	135,933	**1.023 (1.005–1.042)**	**0.014**	135,933	**1.043 (1.024–1.062)**	**<0.001**
Multivariable model (continuous)^a^	135,933	1.000 (0.980–1.020)	0.988	135,933	**1.025 (1.006–1.045)**	**0.010**
Multivariable model (categorical)^b^						
[min, −2 SD)	1629	1.094 (0.931–1.284)	0.275	1549	1.005 (0.852–1.186)	0.951
[−2 SD, −1 SD)	18,094	1.032 (0.978–1.090)	0.250	18,085	0.992 (0.939–1.047)	0.764
[−1 SD, 1 SD]	96,846	Ref.	—	97,160	Ref.	—
(1 SD, 2 SD]	15,013	0.990 (0.933–1.052)	0.75	14,725	1.026 (0.966–1.089)	0.405
(2 SD, max]	4351	**1.115 (1.000–1.223)**	**0.049**	4414	**1.180 (1.068–1.303)**	**0.001**
Levine method						
Age adjusted model (continuous)^a^	135,933	**1.019 (1.001–1.038)**	**0.040**	135,933	**1.044 (1.026–1.063)**	**<0.001**
Multivariable model (continuous)^a^, ^b^	135,933	1.000 (0.981–1.020)	0.963	135,933	**1.035 (1.016–1.054)**	**<0.001**
Multivariable model (categorical)^b^						
[min, −2 SD)	2109	1.122 (0.975–1.291)	0.108	564	0.960 (0.738–1.248)	0.758
[−2 SD, −1 SD)	18,001	1.025 (0.971–1.082)	0.376	16,463	0.987 (0.934–1.043)	0.641
[−1 SD, 1 SD]	96,059	Ref.	—	101,796	Ref.	—
(1 SD, 2 SD]	15,905	0.991 (0.935–1.051)	0.769	12,328	1.057 (0.992–1.126)	0.086
(2 SD, max]	3859	**1.119 (1.006–1.244)**	**0.038**	4782	**1.155 (1.051–1.270)**	**0.003**

Abbreviations: AAge, accelerated age; CI, confidence interval; KDM, Klemera–Doubal method; SD, standard deviation.

^a^ Hazard ratio per 1 SD increase.

^b^ Age was used as timescale with further adjustment for assessment center, Townsend deprivation index, college/university degree, ethnicity, body mass index, smoking status, alcohol status, physical activity, sedentary status, sleep status, diet status, and testosterone.

The bold values are used to indicate statistical significance at type I error of 0.05.

In subgroup analysis for AAge (new method), no statistically significant interactions between categorical CAge and testosterone and AAge were found (Table 4). However, when comparing the AAge at (>2 SD) group with the (−1 SD to 1 SD) group, a significant increase in the risk of BPH was found for PhenoAge‐AAge. Specifically, the HR was 1.904 (95% CI, 1.374–2.639) in the CAge <50 years' subgroup and 1.233 (95% CI, 1.088–1.397) in the testosterone <12 nmol/L subgroup. Furthermore, KDM‐AAge reported similar findings with marginal significance in the two subgroups (both *P* = 0.08). The incidence of BPH increased notably with increasing CAge, regardless of accelerated aging levels. However, only in the CAge <50 years group, when accelerated aging levels rose above 2 SD, the incidence of BPH showed a markedly increased trend (Figure S3). We also found a marginally significant modification effect of AAge on the association between CAge and risk of BPH (Figure S4). Trends for the HRs over CAge were similar for the three PhenoAge‐AAge groups. However, the magnitude of HRs in the >2 SD group was elevated compared to those in both <−2 SD group and −2 SD to 2 SD group.

**TABLE 4:** Subgroup analysis for association between accelerated aging and risk of incident BPH at follow‐up in UK Biobank.

Subgroup	KDM‐AAge (new method)		PhenoAge‐AAge (new method)	
	Hazard ratio (95% CI)	*P*	Hazard ratio (95% CI)	*P*
Age		0.405^a^		0.192^a^
<50 years^b^				
[min, −2 SD)	1.083 (0.560–2.097)	0.812	1.356 (0.725–2.537)	0.341
[−2 SD, −1 SD)	0.978 (0.783–1.223)	0.848	1.052 (0.850–1.302)	0.640
[−1 SD, 1 SD]	Ref.		Ref.	
(1 SD, 2 SD]	1.134 (0.914–1.408)	0.253	1.113 (0.889–1.393)	0.350
(2 SD, max]	1.389 (0.963–2.003)	0.079	**1.904 (1.374–2.639)**	**<0.001**
50 ~ 60 years				
[min, −2 SD)	1.139 (0.847–1.533)	0.390	0.915 (0.645–1.299)	0.620
[−2 SD, −1 SD)	1.077 (0.972–1.194)	0.158	0.982 (0.886–1.089)	0.736
[−1 SD, 1 SD]	Ref.		Ref.	
(1 SD, 2 SD]	1.034 (0.925–1.155)	0.560	1.031 (0.921–1.154)	0.593
(2 SD, max]	0.996 (0.816–1.217)	0.971	1.177 (0.972–1.427)	0.096
>60 years^c^				
[min, −2 SD)	1.079 (0.884–1.318)	0.454	1.018 (0.835–1.240)	0.863
[−2 SD, −1 SD)	1.024 (0.958–1.095)	0.478	0.995 (0.931–1.064)	0.880
[−1 SD, 1 SD]	Ref.		Ref.	
(1 SD, 2 SD]	0.954 (0.885–1.029)	0.220	1.005 (0.933–1.082)	0.900
(2 SD, max]	1.106 (0.977–1.251)	0.113	1.085 (0.958–1.228)	0.199
Testosterone		0.130^a^		0.608^d^
<12 nmol/L				
[min, −2 SD)	1.082 (0.834–1.403)	0.553	1.023 (0.806–1.298)	0.852
[−2 SD, −1 SD)	1.033 (0.953–1.119)	0.434	0.982 (0.911–1.058)	0.628
[−1 SD, 1 SD]	Ref.		Ref.	
(1 SD, 2 SD]	0.929 (0.861–1.001)	0.054	1.006 (0.930–1.089)	0.875
(2 SD, max]	1.111 (0.988–1.249)	0.079	**1.233 (1.088–1.397)**	**0.001**
≥12 nmol/L				
[min, −2 SD)	1.099 (0.895–1.349)	0.368	0.986 (0.783–1.243)	0.908
[−2 SD, −1 SD)	1.033 (0.960–1.112)	0.389	1.001 (0.926–1.083)	0.972
[−1 SD, 1 SD]	Ref.		Ref.	
(1 SD, 2 SD]	1.109 (1.005–1.223)	0.040	1.055 (0.963–1.156)	0.252
(2 SD, max]	1.052 (0.859–1.288)	0.625	1.099 (0.934–1.295)	0.256

*Note*: In the age subgroups, covariates except age were adjustment, including assessment center, Townsend deprivation index, college/university degree, ethnicity, body mass index, smoking status, alcohol status, physical activity, sedentary status, sleep status, diet status, and testosterone. In the testosterone subgroups, age was used as timescale with further adjustment for assessment center, Townsend deprivation index, college/university degree, ethnicity, body mass index, smoking status, alcohol status, physical activity, sedentary status, sleep status, and diet status.

Abbreviations: AAge, accelerated age; KDM, Klemera–Doubal method; SD, standard deviation.

^a^ *p* value for interaction term using likelihood ratio test.

^b^ Four subjects under the age of 40, the minimal age is 38.

^c^ Four subjects over the age of 70, the maximal age is 73.

The bold values are used to indicate statistical significance at type I error of 0.05.

### 3.3 Joint effects of biological age accelerations and genetic susceptibility

**FIGURE 4:**
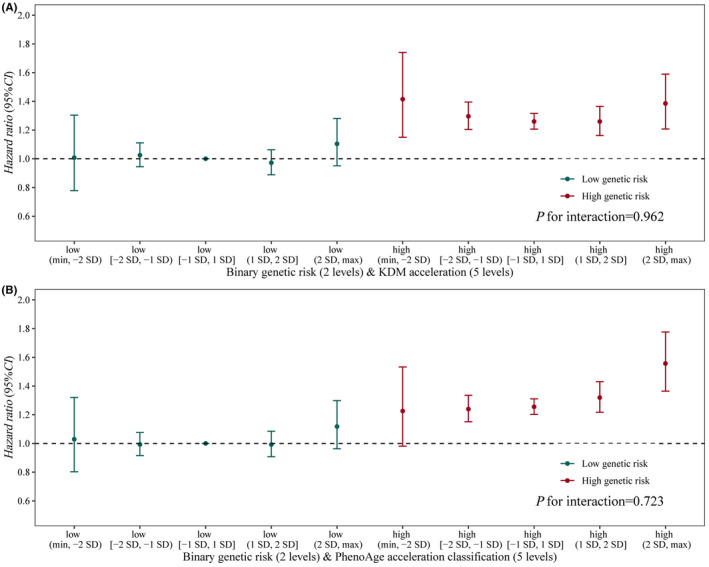
Joint associations of genetic risk and biological age accelerations with the risk of incident BPH at follow‐up.

Calculated PRS was linearly associated with risk of incident BPH, HR = 6.869 (95% CI, 5.323–8.863), *p* for nonlinearity = 0.896 (Figure S5). Participants with higher PRS were more likely to have incident BPH during follow‐up. We evaluated risk synergy between genetic risk and AAge. However, participants' genetic risk did not interact with either KDM‐AAge (*P* = 0.962) or PhenoAge‐AAge (*p* = 0.723). The joint effects of genetic risk and AAge are plotted in Figure 4. Participants with the highest levels of both genetic risk and AAge (>2 SD) presented the highest risk of incident BPH during follow‐up (HR = 1.385 for KDM‐AAge [95% CI, 1.207–1.589], and 1.556 for PhenoAge‐AAge [95% CI, 1.364–1.776]) compared to those with low genetic risk and AAge (−1 SD to 1 SD). Additionally, in the low genetic risk group, neither KDM‐AAge nor PhenoAge‐AAge was associated with the risk of BPH, while statistical significance was observed in the high genetic risk group (Figure 4).

## 4 DISCUSSION

In this prospective study, we observed that both advanced CAge and five biomarker‐based BAge measures were associated with an increased risk of developing BPH, showing a threshold effect at a later stage. Moreover, elevated PhenoAge‐AAge was also linked to an increased risk of incident BPH, and a synergistic effect between CAge and PhenoAge‐AAge was observed. Importantly, we identified a subgroup of participants (40–50 years old) with >2 SD increase in PhenoAge‐AAge who had an approximately twofold increased risk for BPH, compared to peers with balanced AAge (corresponding to −1 SD to 1 SD increase in PhenoAge‐AAge). Even though no evidence supported the interaction effect between the AAge and genetic risk of BPH, higher risk of BPH was still observed in PhenoAge‐AAge with >2 SD and high genetic risk group. These findings suggested BAge and derived AAge were promising biomarkers in midlife and older males who will suffer from BPH in future, independent of CAge and common risk factors.

The global incident cases and disability‐adjusted life‐years (DALYs) of BPH have risen by 105% and 110% from 1990 to 2019, resulting in a substantial disease burden.^25^ A growing number of studies investigated the association of aging biomarkers with BPH. A case–control study showed that shorter telomeres increased the risk of BPH^26^; another study reported DNA methylation (a well‐established aging biomarker) to be associated with BPH.^27^ In this study, we found the associations of CAge, biomarker‐based Bage, and AAge with the incidence of BPH; the effect of CAge on incidence of BPH was also stated in a large global study in 2022.^28^ Individuals with the same CAge can vary substantially in their health and physiological functions. BAge is a direct measure of age‐related physiological changes. It can capture the inter‐individual differences and predict various health outcomes.^13^ The components for calculating BAge in this study were health‐related biomarkers which are routinely obtained during clinical care and health research. Additionally, acquisition of the clinical indicators is more cost‐effective when compared to omics‐based indicators.^29^ Components have been identified as independent risk factor of BPH, such as waist circumference,^30^ and HbA1c.^31^ Moreover, individuals with older BAge than expected for their CAge (AAge > 0) have premature aging and elevated risk of disease, including depressions^19^ and cancers.^6^ Conversely, those with younger BAge (AAge < 0) have delayed aging and greater resilience. CAge moves inexorably forward; BAge can increase, decrease, or stay the same over time depending on environmental and lifestyle factors. This makes BAge a promising target for prediction, prevention, and interventions.

The mechanisms underlying the associations were not investigated for different stages of the prostate aging processes. Molecular alterations associated with biological aging directly influence the physiological processes of the prostate. First, the aging process can lead to cellular senescence, wherein senescent cells remain metabolically active and secrete a range of inflammatory mediators known as the senescence‐associated secretory phenotype (SASP). This SASP was associated with the initiation and progression of BPH.^29^ The SASP‐derived secondary aging disrupts tissue homeostasis, leading to loss of tissue repair and regeneration in both proximal and distal modes.^32^ SASP also helps to maintain and enhance inflammation, resulting in raised chronic and low‐grade systemic inflammation.^33^ Second, oxidative stress is another cellular condition associated with age. As individuals age, the balance between pro‐oxidants and antioxidants within cells tends to shift toward a more oxidizing state, thereby disrupting the body's redox homeostasis. Individuals with accelerated biological aging often exhibit higher levels of oxidative stress markers when compared to their Cage‐matched peers with normal biological aging.^34^, ^35^ Elevated levels of reactive oxygen species diminish the antioxidant defense capacity of the body, resulting in damage to proteins, lipids, and DNA and impairment of cellular function, contributing to the initiation and development of BPH.^36^

The BAge and AAge aim to offer a comprehensive assessment of an individual's aging process by capturing variations in resilience and physiological dysfunction. Although the risk of BPH increases with age, improving biological age might delay BPH in the elderly. Importantly, BAge based on clinical biomarkers is modifiable. Slowing biological aging can be a key goal of proactive prevention and anti‐aging intervention for BPH, as it has the potential to enhance the quality of life for older males and result in significant health care cost savings. Moreover, BAge measured from biomarkers allows researchers to test if an intervention affects the fundamental biology of aging. In a pilot randomized controlled clinical trial, the implementation of a dietary and lifestyle program led to an average reversal of BAge of 3.23 years compared to the control group.^37^ It suggested that a diet and lifestyle intervention might have a favorably impact on BAge during midlife and beyond. We observed a higher risk of BPH associated with advanced AAge in subgroups of participants aged 40–50 years old and those with a high genetic risk. Individuals with BAge acceleration across the first half of the lifespan were more prone to develop BPH. It might be because younger adults are more sensitive to risk exposures that shorten healthy lifespan.^13^ Active health interventions at an early age are more urgent for preventing BPH. The impact of BAge acceleration can influence the development of BPH independently of genetic risk; however, males with high genetic risk should actively improve their BAge. More attention should also be given to the nongenetic‐driven etiology studies of BPH.^38^

There are several limitations to be considered in this study. First, there is no “gold standard” method for measuring aging, especially for the prostate. Different biomarkers and algorithms used in this study have captured different aspects of aging and come to less than consistent conclusions. More importantly, the UK biobank lacks comprehensive data on prostate aging and the severity of BPH, including parameters such as the International Prostate Symptom Score (IPSS), limiting our ability to thoroughly evaluate the relationship between advanced biological aging levels and various stages of BPH. As a result, our study might not fully capture the entire spectrum of prostate aging and its progression. Given that the prostate aging and BPH can be divided into four clinical stages^11^, ^39^; hence, we suggest performing studies that incorporate detailed biomarkers and clinical symptom‐based scores at all distinct stages of prostate aging, instead of solely focusing on the binary BPH outcome, to provide a more comprehensive understanding of the relationship. Second, only a subset of UK Biobank participants completed the follow‐up surveys, BAge and AAge were measured only at baseline, so the study could not analyze changes in AAge over time in relation to changes in risk of BPH. Third, the UK Biobank sample might be healthier and wealthier than the general population, the current study consisted predominantly of middle‐aged and older white adults, which might limit the generalizability of our results. We suggest performing studies across the general population. Fourth, the study was observational, despite careful adjustments for various BPH risk factors, causality could not be definitively established. Meanwhile, even though it was a large sample size study with a long follow‐up, the substantial amount of missing data for the calculation of biological age might lead to bias in the association. High‐quality randomized controlled trials should be conducted. Recently, a study established animal model of heterochronic parabiosis and found that young blood induced “rejuvenation” in aged individuals and old blood accelerated “aging” in young individuals^40^; hence, animal experiments exploring the prostate aging and advanced BAge could be conducted to provide comprehensive evidence. Lastly, although the Cox model is a commonly used method for time‐to‐event outcome and estimated HR provides a relative effect measure, they may not always be the optimal presentation. Further studies utilizing multiple statistical methods could provide more comprehensive effect measures. For instance, parametric accelerated failure time (AFT) models can provide survival time ratio (acceleration factor), and restricted mean survival time‐based methods can offer differences in restricted mean survival time (absolute effect) and ratio of restricted mean survival time (relative effect).

## 5 CONCLUSIONS

In summary, our findings indicated that BAge and its acceleration might be an independent and modifiable risk factor for incident BPH, particularly among males aged 40–50 years. Beginning from midlife, active health interventions to slow biological aging, including physical exercise and healthy diet, may help mitigate the detrimental effects of accelerated aging and reduce the burden of disease.

## AUTHOR CONTRIBUTIONS

X.‐T.Z., J.H., and Y.‐H.J. contributed to study design. Q.H., B.‐H.L., and Y.‐B.W. contributed to data collection. Q.H., Y.‐B.W., and H.Z. contributed to data analysis, H.Z., Y.‐Y.Z., F.L., C.F., and S.‐D.T. contributed to data interpretation. Q.H. and J.H. wrote the manuscript. All authors read and approved the final manuscript.

## FUNDING INFORMATION

This work was supported (in part) by the National Key Research and Development Program of China (Grant No. 2022YFC3600700, Prof. Xian‐Tao Zeng), the Young Top‐notch Talent Cultivation Program of Hubei Province (Prof. Xian‐Tao Zeng), and the Program of Excellent Doctoral (Postdoctoral) of Zhongnan Hospital of Wuhan University (Grant No. ZNYB2021044, Dr Jiao Huang).

## CONFLICT OF INTEREST STATEMENT

The authors declare that they have no conflict of interest.

## ETHICS STATEMENT

The UK Biobank is a large‐scale and continuously evolving longitudinal cohort study which was approved by the UK North West Multicenter Research Ethical Committee. Prior to participation, all individuals provided written informed consent.

## Supporting information


Figures S1–S5

Tables S1–S5
