# Supplementary material for: Clinical biomarker‐based biological aging and risk of benign prostatic hyperplasia: A large prospective cohort study
Source: Aging Med (Milton). 2024 Jun 14;7(3):393–405. doi: 10.1002/agm2.12331 (PMC11222739; doi:10.1002/agm2.12331)
Supplement: Supplementary file 1 — Figures S1–S5 [file AGM2-7-393-s002.docx]

**Supplementary Figures**

Figure S1. Flowchart of participant selection

Figure S2. Biological age measures

Figure S3. Incidence of BPH in different age and accelerated age groups

Figure S4. Chronological age and risk of incident BPH stratified by accelerated age groups

Figure S5. Joint effect of polygenic risk score and accelerated age on risk of incident BPH at follow-up





Figure S1. Flowchart of participant selection


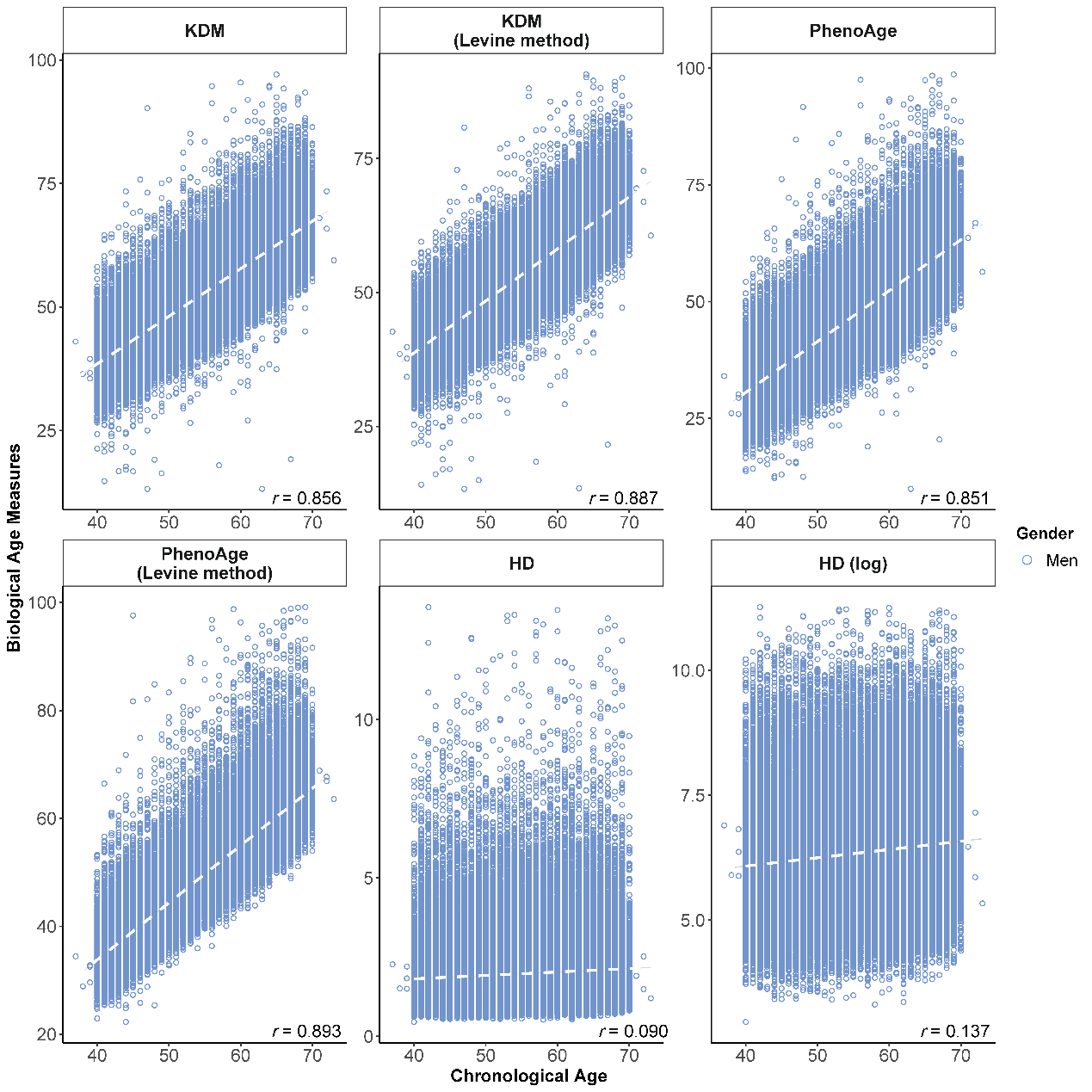


Figure S2. Biological age measures


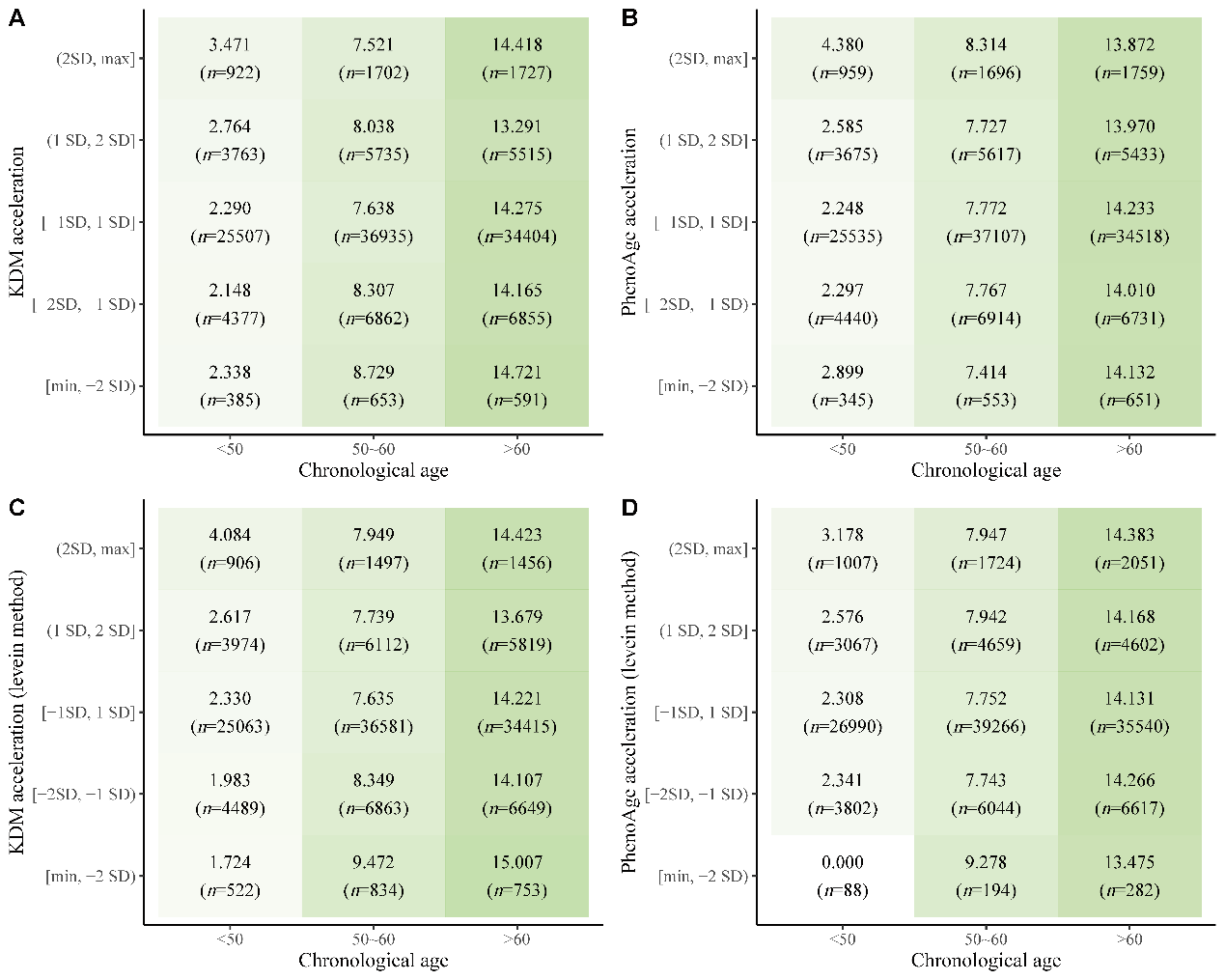


Figure S3. Incidence of BPH in different age and accelerated age groups


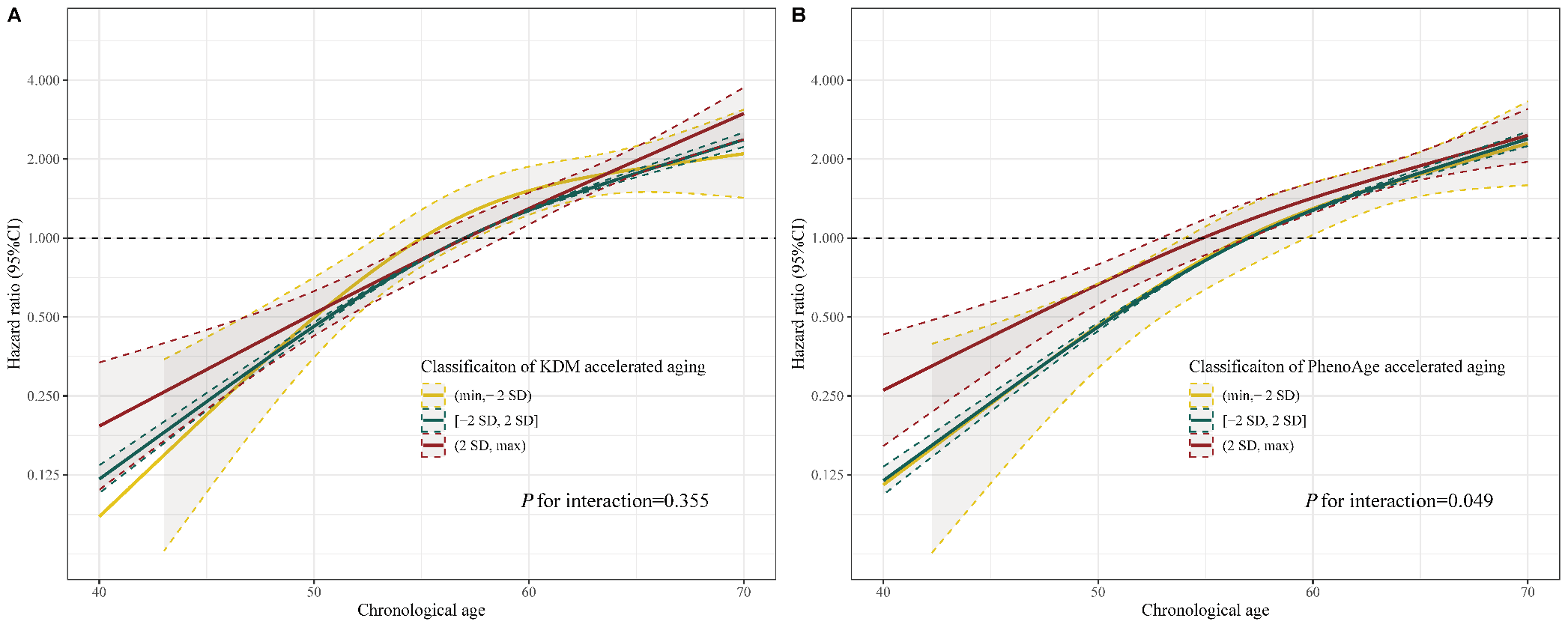


Figure S4. Chronological age and risk of incident BPH stratified by accelerated age groups


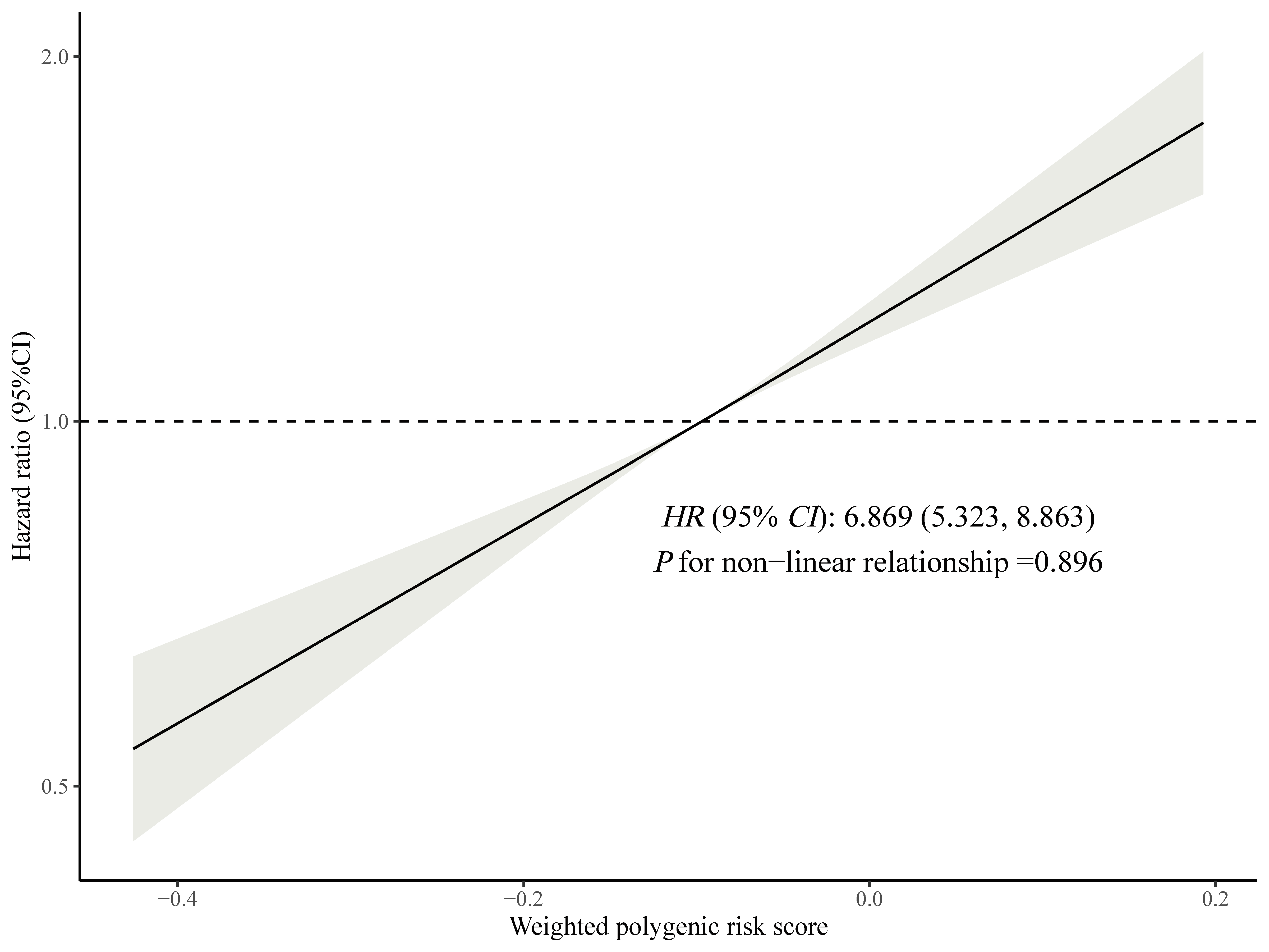


Figure S5. Joint effect of polygenic risk score and accelerated age on risk of incident BPH at follow-up
