# Supplementary material for: Clinical biomarker‐based biological aging and risk of benign prostatic hyperplasia: A large prospective cohort study
Source: Aging Med (Milton). 2024 Jun 14;7(3):393–405. doi: 10.1002/agm2.12331 (PMC11222739; doi:10.1002/agm2.12331)
Supplement: Supplementary file 2 — Tables S1–S5 [file AGM2-7-393-s001.docx]

**Supplementary Tables**

Table S1. Biomarkers included in the biological age algorithms.

Table S2. Measurement methods of Biomarkers in the biological age algorithms from UK Biobank.

Table S3. 19 independent single nucleotide polymorphisms (SNPs) associated (P<5e-8) with BPH.

Table S4. Definition and determination of included covariates.

Table S5. Associations between individual clinical biomarkers and risk of BPH in UK Biobank

**Table S1. Biomarkers included in the biological age algorithms.**

| Biomarkers | Correlation with CA  in NHANES III (male) | Included in new method  of KDM, PhenoAge, and HD | Included in KDM  (Levine method) | Included in PhenoAge  (Levine method) |
| --- | --- | --- | --- | --- |
| FEV_1_ (L) | -0.711 | 🗸 | 🗸 |  |
| Waist circumference (cm) | 0.308 | 🗸 |  |  |
| Total cholesterol (mg/dL) | 0.194 | 🗸 | 🗸 |  |
| Triglyceride (mg/dL) | 0.105 | 🗸 |  |  |
| SBP (mm Hg) | 0.509 | 🗸 | 🗸 |  |
| DBP (mm Hg) | 0.060 |  |  |  |
| HbA1c (%) | 0.296 | 🗸 | 🗸 |  |
| Serum glucose (mmol/L) | 0.243 | 🗸 |  | 🗸 |
| Blood urea nitrogen (mg/dL) | 0.386 | 🗸 | 🗸 |  |
| C-reactive protein (mg/dL) | 0.189 | 🗸 | 🗸 | 🗸 |
| Creatinine (μmol/L) | 0.305 | 🗸 | 🗸 | 🗸 |
| Albumin (g/dL) | -0.398 | 🗸 | 🗸 | 🗸 |
| Alkaline phosphatase (U/L) | 0.106 | 🗸 | 🗸 | 🗸 |
| Uric acid (mg/dL) | 0.026 |  |  |  |
| Red cell distribution width (%) | 0.355 | 🗸 |  | 🗸 |
| Mean cell volume (fL) | 0.185 | 🗸 |  | 🗸 |
| Lymphocyte (%) | -0.206 | 🗸 |  | 🗸 |
| RBC count (million cells/μL) | -0.355 | 🗸 |  |  |
| WBC count (1000 cells/μL) | 0.042 |  |  | 🗸 |
| Number of included biomarkers | 18+1 | 16 | 9 | 9 |
| NHANES, National Health and Nutrition Examination Survey; CA, chronological age; KDM, Klemera-Doubal method; HD, homeostatic dysregulation; FEV1, forced expiratory volume in 1 second; SBP, systolic blood pressure; DBP, diastolic blood pressure; HbA1c, glycated hemoglobin; RBC, red blood cell; WBC, white blood cell. | | | | |

**Table S2. Measurement methods of Biomarkers in the biological age algorithms from UK Biobank.**

| Biomarkers | Source name  in UK biobank | Measurement method  in UK Biobank |
| --- | --- | --- |
| FEV1 (L) | Forced expiratory volume in 1-second (FEV1) | Breath spirometry is performed using a Vitalograph Pneumotrac 6800 (Vitalograph, UK; ) operated via a PC for data capture and in order to visualise flow graphs from each test. |
| Waist circumference (cm) | Waist circumference | Find the bottom of your ribs and the top of your hips. Place a tape measure around your middle at a point halfway between them (just above the belly button). Make sure it's pulled tight, but isn't digging into your skin. Breathe out naturally and take your measurement. |
| Total cholesterol (mg/dL) | Total cholesterol | Measured by CHO-POD analysis on a Beckman Coulter AU5800 |
| Triglyceride (mg/dL) | Triglycerides | Measured by GPO-POD analysis on a Beckman Coulter AU5800 |
| SBP (mm Hg) / DBP (mm Hg) | SBP / DBP | Blood pressure, manual reading, systolic. Two measures of blood pressure were taken a few moments apart. A manual sphygmometer was used if the standard automated device could not be employed. A manual sphygmometer was used if the standard automated device could not be employed. |
| HbA1c (%) | HbA1c | Measured by HPLC analysis on a Bio-Rad VARIANT II Turbo |
| Serum glucose (mmol/L) | Glucose | Measured by hexokinase analysis on a Beckman Coulter AU5800 |
| Blood urea nitrogen (mg/dL) | Urea | Measured by GLDH, kinetic analysis on a Beckman Coulter AU5800 |
| C-reactive protein (mg/dL) | C-reactive protein | Measured by immunoturbidimetric - high sensitivity analysis on a Beckman Coulter AU5800 |
| Creatinine (μmol/L) | Creatinine | Measured by enzymatic analysis on a Beckman Coulter AU5800 |
| Albumin (g/dL) | Albumin | Measured by BCG analysis on a Beckman Coulter AU5800 |
| Alkaline phosphatase (U/L) | Alkaline phosphatase | Measured by AMP(IFCC) analysis on a Beckman Coulter AU5800 |
| Uric acid (mg/dL) | Urate | Measured by uricase PAP analysis on a Beckman Coulter AU5800 |
| Red cell distribution width (%) | Red blood cell (erythrocyte) distribution width | Result of "Red Distribution Width" assay, performed on blood sample, obtained from UK Biobank assessment centre visit. Red blood cell Distribution Width is the size distribution spread of the erythrocyte population derived from the red blood cell histogram. It is the coefficient of variation (CV) expressed in % of the red blood cell size distribution. |
| Mean cell volume (fL) | Mean corpuscular volume | Result of "Mean Corpuscular Volume" assay, performed on blood sample, obtained from UK Biobank assessment centre visit. Mean Corpuscular Haemoglobin Concentration (pg) is the weight of hemoglobin in the average erythrocyte, computed by the formula: MCH = (hemoglobin/red blood cells) x 10. |
| Lymphocyte (%) | Lymphocyte percentage | Result of "Lymphocytes Percentage" assay, performed on blood sample, obtained from UK Biobank assessment centre visit. Lymphocytes (Percentage) is calculated as the proportion of lymphocytes in the leukocytes. |
| RBC count (million cells/μL) | RBC count | Four Beckman Coulter LH750 instruments were utilised to analyse samples collected in 4ml EDTA (Ethylenediaminetetraacetic acid) vacutainers for the (circa) 500,000 participants of the UK Biobank baseline cohort |
| WBC count (1000 cells/μL) | WBC count | Four Beckman Coulter LH750 instruments were utilised to analyse samples collected in 4ml EDTA (Ethylenediaminetetraacetic acid) vacutainers for the (circa) 500,000 participants of the UK Biobank baseline cohort |

FEV1, forced expiratory volume in 1 second; SBP, systolic blood pressure; DBP, diastolic blood pressure; HbA1c, glycated hemoglobin; RBC, red blood cell; WBC, white blood cell.

**Table S3. 19 independent single nucleotide polymorphisms (SNPs) associated (P<5e-8) with BPH.**

| Source | rsid | chromosome | chromosome position | effect allele | non-effect allele | beta |
| --- | --- | --- | --- | --- | --- | --- |
| Gudmundsson, J. et al. [1] | rs2556378 | 2 | 60762502 | T | G | 0.049218023 |
|  | rs2853677 | 5 | 1287194 | G | A | 0.037426498 |
|  | rs381949 | 5 | 1322468 | G | A | 0.045322979 |
|  | rs10054105 | 5 | 110909333 | T | G | 0.041392685 |
|  | rs677394 | 5 | 134607559 | C | G | 0.056904851 |
|  | rs200476 | 6 | 27768348 | A | T | 0.056904851 |
|  | rs148678804 | 10 | 22427289 | A | G | 0.103803721 |
|  | rs4548546 | 10 | 122629579 | T | C | 0.045322979 |
|  | rs11199879 | 10 | 123045212 | C | T | 0.056904851 |
|  | rs72878024 | 11 | 199492 | G | A | 0.071882007 |
|  | rs2555019 | 12 | 114668618 | C | T | 0.033423755 |
|  | rs8853 | 12 | 115108907 | C | T | 0.029383778 |
|  | rs1638703 | 13 | 51088356 | C | G | 0.041392685 |
|  | rs11651052 | 17 | 36102381 | G | A | 0.033423755 |
|  | rs9958656 | 18 | 19904144 | T | C | 0.045322979 |
| W. Li et al. [2] | rs8027714 | 15 | 24964597 | A | G | -0.180456064 |
|  | rs8136152 | 22 | 43776080 | G | A | -0.142667504 |
|  | rs10192133 | 2 | 161475895 | C | T | 0.11058971 |
|  | rs1237696 | 11 | 100917073 | T | C | 0.133538908 |

[1] Gudmundsson, J., et al. Genome-wide associations for benign prostatic hyperplasia reveal a genetic correlation with serum levels of PSA. Nat Commun 9, 4568 (2018).

[2] Li, W. & Klein, R.J. Genome-wide association study identifies a role for the progesterone receptor in benign prostatic hyperplasia risk. Prostate Cancer Prostatic Dis 24, 492-498 (2021).

**Table S4. Definition and determination of included covariates**

| Covariates | Field ID | Definition and recode |
| --- | --- | --- |
| Age | 21022 | Age at recruitment |
| Assessment centers | 54 | England (Barts, Birmingham, Bristol, Bury, Cheadle, Croydon, Hounslow, Leeds, Liverpool, Manchester, Newcastle, Nottingham, Oxford, Reading, Sheffield, Stockport, Stoke)  Wales (Cardiff, Swansea, Wrexham)  Scotland (Edinburgh, Glasgow) |
| Ethnicity | 21000 | White (1, 1001, 1002, 1003)  Asian (3, 3001, 3002, 3003, 3004, 5)  Black (4, 4001, 4002, 4003)  Others (2, 2001, 2002, 2003, 2004, 6) |
| Townsend deprivation index | 22189 | Townsend deprivation index at recruitment |
| College/university degree | 6138 | Yes (1)  No (2, 3, 4, 5, 6, -7) |
| Body mass index | 21001 | Body mass index at recruitment |
| Testosterone | 30850 | Testosterone |
| Smoking status | 20116 | Never (0)  Previous (1)  Current (2) |
| Alcohol status | 20117 | Never (0)  Previous (1)  Current (2) |
| Regular physical activity status ^[1]^ | 884, 894, 904,  914 | Moderate activity = Number of days/week of moderate physical * Duration of moderate activity  Vigorous activity = Number of days/week of vigorous physical * Duration of vigorous activity  **Classification:**  **Ideal (Moderate activity per week ≥15 or Vigorous activity per week ≥ 7 or Moderate activity per week + Vigorous activity per week ≥ 150)**  **Intermediate/poor (Total score >1)**  **Unknown** |
| Sedentary status ^[2]^ | 1080, 1070 | **Total score= Time spent using computer+ Time spent watching television (TV)**  **Classification:**  **Ideal (Total <=1)**  **Intermediate/poor (Total score >1)**  **Unknown** |
| Sleep status ^[3]^ | 1160, 1170, 1180, 1200, 1210, 1220 | Sleep duration: 1 for low risk (7-8 hours) and 0 for high risk (others);  Morning routine: 1 for 1 for low risk (easy) and 0 for high risk (hard);  Chronotype: 1 for low risk ('morning' person) and 0 for high risk ('evening' person);  Sleeplessness/insomnia: 1 for low risk (Never/rarely) and 0 for high risk (Sometimes/usually);  Snoring: 1 for low risk (No) and 0 for high risk (Yes);  Daytime dozing/sleeping: 1 for low risk (Never/rarely/Sometimes) and 0 for high risk (Often/ All of the time);  **Total score = morning routine + chronotype + Sleeplessness/insomnia + snoring + daytime dozing or sleeping**  **Classification:**  **Ideal (Total >=5)**  **Intermediate/poor (Total score <=4)**  **Unknown** |
| Diet status ^[1]^ | 1289,  1299, 1309, 1319, 1329, 1339, 1349, 1369, 1379, 1389 | Total fruit intake (1-healthy/0-Unhealthy) = Fresh fruit intake + Dried fruit intake  Total vegetable intake (1-healthy/0-Unhealthy) =Cooked vegetable intake + Salad or raw vegetable intake  Total fish intake (1-healthy/0-Unhealthy) = Oily fish intake + Non oily fish intake  Processed and red meat intake (1-healthy/0-Unhealthy) =Processed meat intake + Beef intake + Lamb or mutton intake + Pork intake  Total score = Total fruit intake + Total vegetable intake + Total fish intake + Processed and red meat intake  **Classification:**  **Ideal (Total >=3)**  **Intermediate/poor (Total score <3)**  **Unknown** |

[1] Shen M, Xiao Y, Jing D, et al. Associations of combined lifestyle and genetic risks with incident psoriasis: A prospective cohort study among UK Biobank participants of European ancestry. J Am Acad Dermatol. 2022;87(2):343-350. doi:10.1016/j.jaad.2022.04.006

[2] Kim, Y., Yeung, S.L.A., Sharp, S.J. et al. Genetic susceptibility, screen-based sedentary activities and incidence of coronary heart disease. BMC Med 20, 188 (2022). <https://doi.org/10.1186/s12916-022-02380-7>

[3] Gao X, Huang N, Guo X, Huang T. Role of sleep quality in the acceleration of biological aging and its potential for preventive interaction on air pollution insults: Findings from the UK Biobank cohort. Aging Cell. 2022;21(5):e13610. doi:10.1111/acel.13610

**Table S5. Associations between individual clinical biomarkers and risk of BPH in UK Biobank**

| Biomarkers | coefficient | HR (95% confidence interval) | P |
| --- | --- | --- | --- |
| FEV_1_ (L) | -0.0567 | 0.945 (0.919, 0.971) | <0.001 |
| Waist circumference (cm) | 0.0087 | 1.009 (1.006, 1.011) | <0.001 |
| Total cholesterol (mg/dL) | -0.0019 | 0.998 (0.998, 0.998) | <0.001 |
| Triglyceride (mg/dL) | -0.0001 | 1.000 (1.000, 1.000) | 0.264 |
| SBP (mm Hg) | -0.0017 | 0.998 (0.997, 0.999) | <0.001 |
| HbA1c (%) | 0.0484 | 1.050 (1.022, 1.078) | <0.001 |
| Serum glucose (mmol/L) | 0.0003 | 1.000 (0.987, 1.014) | 0.970 |
| Blood urea nitrogen (mg/dL) | 0.0150 | 1.015 (1.011, 1.020) | <0.001 |
| C-reactive protein (mg/dL) | 0.0243 | 1.025 (0.981, 1.071) | 0.277 |
| Creatinine (μmol/L) | 0.0014 | 1.001 (1.000, 1.003) | 0.019 |
| Albumin (g/dL) | -0.0276 | 0.973 (0.966, 0.980) | <0.001 |
| Alkaline phosphatase (U/L) | 0.0007 | 1.001 (1.000, 1.001) | 0.060 |
| Red cell distribution width (%) | 0.0505 | 1.052 (1.031, 1.073) | <0.001 |
| Mean cell volume (fL) | -0.0121 | 0.988 (0.984, 0.991) | <0.001 |
| Lymphocyte (%) | -0.0096 | 0.990 (0.988, 0.993) | <0.001 |
| RBC count (million cells/μL) | 0.1510 | 1.163 (1.107, 1.222) | <0.001 |
| WBC count (1000 cells/μL) | 0.0195 | 1.020 (1.010, 1.029) | <0.001 |

FEV1, forced expiratory volume in 1 second; SBP, systolic blood pressure; DBP, diastolic blood pressure; HbA1c, glycated hemoglobin; RBC, red blood cell; WBC, white blood cell. All biomarkers were standardized with mean=0 and standard deviation=1, and HRs were standard deviation increase in the biomarker level. All models were adjusted for assessment center, Townsend deprivation index, college/university degree, ethnicity, body mass index, smoking status, alcohol status, physical activity, sedentary status, sleep status, diet status and testosterone.
